# Characteristics of intestinal-related lymphoid hyperplasia in children and its correlation with intussusception of children

**DOI:** 10.1186/s12887-022-03675-7

**Published:** 2022-11-05

**Authors:** Qingtao Yan, Liandi Xu, Jun Chao, Zemin Zhang, Hui Wang

**Affiliations:** 1 Department of Pediatric Surgery, Weifang People’s Hospital, 151 Guangwen St, Kuiwen District, 261041 Weifang, China; 2 Department of Ultrasound, Weifang People’s Hospital, 261041 Weifang, China; 3 Department of Pediatric Surgery, Weifang Medical College, 261503 Weifang, China; 4 Department of Dermatology, Weifang People’s Hospital, 261041 Weifang, China

**Keywords:** Intussusception in children, Intestinal-associated lymphoid tissue, Intestinal wall lymphatic tissue, Mesenteric lymphatic tissue, Right lower abdominal total mesenteric lymph node volume(RLTMLNV), Periumbilical total mesenteric lymph node volume (PTMLNV), Hyperplasia of intestinal associated lymphoid tissue

## Abstract

**Background:**

Primary intussusception in children is a common acute abdominal disease. The cause of this disease is still not fully understood. Many articles have reported that children with intussusception are often accompanied by hyperplasia of mesenteric lymph nodes and submucosal lymphoid tissue of the terminal ileum. Therefore, hyperplasia of intestinal-associated lymphoid tissue (mesenteric lymph nodes and submucosal lymphoid tissue of the intestinal tract) may be one of the main causes of intussusception. However, the characteristics and differences of intestinal-associated lymphoid tissues in healthy children and children with intussusception at different ages have not been reported. In addition, the relationship between mesenteric lymph nodes and intestinal submucosal lymphatic tissue also needs to be further understood.

**Methods:**

73 patients with intussusception during the recovery phase who were treated in our hospital from October 2019 to October 2021 were collected as the observation group, while 101 children with healthy physical examination or diseases unrelated to intestinal lymphoid hyperplasia were collected as the control group. They were divided into different age groups of 1–6 months, 7–12 months, 13–18 months, 19–24 months, 25–36 months, 3–4 years, 5–6 years, and 7–8 years old. Ultrasonography was used to explore and scan mesenteric lymph nodes in fixed areas of the right lower abdomen and around the umbilicus. The size (cm^3^) and number (n) of detectable lymph nodes in each region were recorded and calculated, and the total mesenteric lymph node volume (cm^3^) of the right lower abdomen (RLTMLNV) and periumbilical region (PTMLNV) was calculated, respectively. The total mesenteric lymph node volume of each region in different ages of the two groups was analyzed.

**Results:**

(1) There were significant differences between the control group and the observation group in the right lower abdominal total mesenteric lymph nodes volume (RLTMLNV) and the periumbilical total mesenteric lymph nodes volume (PTMLNV) (P = 0.001). The mesenteric lymph nodes in the observation group showed severe hyperplasia. (2) Children with intussusceptions are usually accompanied by severe mesenteric lymphoid hyperplasia. The mean volume value of RLTMLNV was greater than that of PTMLNV. Especially within 2 years of age, the mean value of RLTMLNV was significantly higher than that of PTMLNV with statistical significance (P < 0.05). (3) In normal children (control group), lymph nodes in the right lower abdomen and periumbilical area showed low hyperplasia, and there was a significant difference between age groups of < 2 years old and 2–8 years old (p = 0.001). In the children with intussusception (observation group), the hyperplasia of mesenteric lymph nodes in the right lower abdomen and around the umbilicus was severe. There was no significant difference in the proliferation of mesenteric lymphoid tissue among different age groups in the right lower abdomen (P = 0.834). There was also no significant difference in hyperplasia of periumbilical mesenteric lymphoid tissue among different age groups (P = 0.097).

**Conclusions:**

Our research shows: (1) The occurrence of primary intussusception in children is related to the hyperplasia of intestinal-associated lymphoid tissue. (2) Children with intussusceptions were usually accompanied by severe mesenteric lymphoid hyperplasia. The mesenteric lymphoid hyperplasia was more evident in the right lower abdominal ileocecal area than in the periumbilical area before 2 years of age. RLTMLNV has better predictability of intussusception than PTMLNV. The occurrence of intussusceptions was more closely related to the hyperplasia of intestinal-associated lymphoid tissue in the right lower abdomen. (3) Normal children showed a low degree of mesenteric lymphoid hyperplasia before 2 years old, moderate hyperplasia after 2 years old, and mesenteric lymphoid hyperplasia in the right lower abdominal ileocecal area was basically the same as the periumbilical area. The lymphatic tissue of the right lower abdomen and periumbilical mesentery in children with intussusceptions showed severe hyperplasia, and there were no significant differences among different age groups.

## Background

Primary intussusception is a common disease in children. It is a characteristic disease of infants. The incidence gradually decreases with age and has an increasing trend at present. Its main manifestations are paroxysms of abdominal pain, sausage-like lump in the abdomen, jam-like bloody stools, and tree rings-like mass in the section of abdominal exploration by color doppler ultrasound. If the treatment is not timely, intestinal perforation, necrosis, and even life-threatening can be caused [1]. Although it is a common disease, its pathogenesis is not well understood[2]. In this paper, intestinal-associated lymphoid tissue refers to intestinal antigen-associated lymphoid tissue [3, 4], which can be divided into two parts. One is intestinal wall lymphoid tissue, including submucosal mass lymphoid tissue and Peyer patches, also known as mucosal-associated lymphoid tissue (MALT). The other is the lymphatic tissue in the mesenteric lymph nodes. They are closely related to the intestinal immune system. The solid antigenic substances in the intestinal lumen were transported into the submucosa by M cells swallowing the antigenic substances in the intestinal mucosa. They met with specific cloned B and T lymphocytes entering the submucosa from the blood via high columnar endothelial venule (HEV). With the assistance of macrophages, dendritic cells and T helper cells, T and B lymphocytes proliferated, resulting in submucosal lymphoid tissue hyperplasia. On the other hand, intestinal antigens that entered the submucosa through M cells and activated T and B lymphocytes can also enter mesenteric lymph nodes through lymphatic transport and output channels, and lymphocyte proliferation causes mesenteric lymph node proliferation. The two parts of the intestinal lymphatic tissue are closely related to the immune response to intestinal antigens. They work together to generate an immune response to intestinal antigens. The same intestinal antigens stimulate both to induce an immune response and develop lymphoid tissue hyperplasia [4]. Lymphoid hyperplasia in the submucosal lymphoid tissue of the intestinal wall and mesenteric lymph nodes should co-occur. Measuring the hyperplasia degree of mesenteric lymph nodes can also indirectly reflect the hyperplasia of intestinal submucosal lymphatic tissue. The right lower abdominal mesenteric lymph node mainly drains intestinal antigens and lymphocytes from the ileocecal bowel, and The periumbilical lymph node mainly drains intestinal antigens and lymphocytes from the intestinal canal except the ileocecal intestinal canal. The hyperplasia of lymph nodes in the mesentery of the right lower abdomen is more likely to reflect the hyperplasia of submucosal lymphoid tissue in the ileocecal terminal ileum. According to articles [5–9], primary intussusceptions in children are closely related to mesenteric lymph node enlargement and lymphoid hyperplasia of the intestinal wall, especially submucosal lymphoid hyperplasia of the terminal ileum. Since it is challenging to detect hyperplasia of submucosal lymphoid tissue at the terminal ileum by current non-invasive detection methods, most cases are from case reports or excised ileocecal specimens, thus resulting in the lack of systematic studies on this theory. Moreover, most articles have not clarified the correlation between the two parts of lymphoid tissue in children’s pathogenesis of primary intussusceptions. Due to the synchronous hyperplasia between intestinal submucosal lymphoid tissue hyperplasia and mesenteric lymph nodes hyperplasia, we can indirectly predict the degree of intestinal wall lymphoid tissue hyperplasia by measuring the degree of mesenteric lymph node hyperplasia by ultrasound, then analyze and observe its correlation with intussusceptions. Some literature [7] used the measurement of mesenteric lymph node length diameter > 1 cm and the ratio of length diameter to transverse diameter > 2 as the basis for the existence of mesenteric lymph node hyperplasia, but it is not easy to quantify. In our study, we will use a new quantifiable method to characterize the degree of mesenteric lymph node proliferation. This method measures the number and volume of each visible mesenteric lymph node in a specific region around the umbilicus and right lower abdomen by ultrasound. After that, we add them together to obtain the total volume of mesenteric lymph nodes in the right lower abdomen (RLTMLNV)and the total volume of mesenteric lymph nodes in the periumbilical area (PTMLNV). They can reflect the extent of mesenteric lymphoid tissue hyperplasia.

## Data and methods

### Clinical data

A total of 73 children in the recovery period of intussusception (2–30 days after reduction of intussusception) who had been successfully reduced by saline enema under ultrasound detection in our hospital since 2019 were collected, including 46 males and 27 females, the age ranged from 3 months to 8 years. These children were set as the observation group. Meanwhile, 101 children, 55 males and 46 females, who were randomly collected from healthy children or children with some diseases unrelated to intestinal lymphoid hyperplasia since 2019, were set as controls. The age ranged from 1 month to 8 years old.

### Methods

The control group and the observation group were divided into different age groups (1–6 months, 7–12 months, 13–18 months, 19–24 months, 25–36 months, 3–4 years old, 5–6 years old, 7–8 years old) for control study. A continuous multi-section abdominal scan was performed with the high-frequency 9 linear array probe of GELOGIQ E9 ultrasonography. The number, long diameter and short diameter (cm) of visible mesenteric lymph nodes around the umbilicus (superior and inferior mesenteric artery distribution area) and right lower abdomen were recorded. Since mesenteric lymph nodes are mostly ellipsoid shaped, the approximate value of each lymph node volume (cm^3^) was obtained according to the ellipsoid volume formula (long diameter x short diameter x short diameter x 3.14 ÷ 6). The volume of each mesenteric lymph node was summed up to obtain the total volume of the right lower abdomen(RLTMLNV)and periumbilical mesenteric lymph nodes (PTMLNV). The mean total volume (cm^3^) of right lower abdominal and periumbilical mesenteric lymph nodes in different age groups was calculated, expressed as Median(interquartile range), and statistically analyzed.

### Statistical methods

SPSS19.0 was used for statistical analysis. The Median (interquartile range) was used for nonnormal distribution measurement data. Mann-Whitney U test was used for the nonparametric test between the two groups. Wilcoxon W test was used for paired data, and the Kruskal-Wallis H test was used for the nonparametric test between multiple groups. The optimum cut-off value for predicting the occurrence of intussusception was found by the receiver operating characteristic curve (ROC), and the area under the receiver operating characteristic curve (AUC) was calculated. p < 0.05 was considered statistically significant.

## Result

### Comparison of RLTMLNV and PTMLNV between the control group and the observation group in different age groups.

The control group consisted of 101 children, 55 males and 46 females. A total of 73 children were included in the observation group, including 46 males and 27 females, as shown in Table 1. The RLTMLNV and PTMLNV in the observation group were significantly higher than in the control group. Compared with the control group, the RLTMLNV was significantly different in all age groups from 0 to 8 years old (P < 0.05) (Table 2). The PTMLNV in the observation group was significantly different from that in the control group in different age groups of 0–4 years old (P < 0.05), but there was no significant difference in 5–8 years old (P > 0.05) (Table 3). The results show that the incidence of intussusception was closely related to the proliferation of intestinal-associated lymphoid tissue (mesenteric lymphoid tissue and intestinal wall lymphoid tissue). In older children (≥ 5 years old), the difference in intestinal-associated lymphoid tissue proliferation between the control group and the observation group decreased, and the p-value increased gradually (Tables 2 and 3), accompanying the incidence of intussusception decreases in this age range.

### Comparison of predictive differences in intussusception between the RLTMLNV and PTMLNV.

**Fig. 1:**
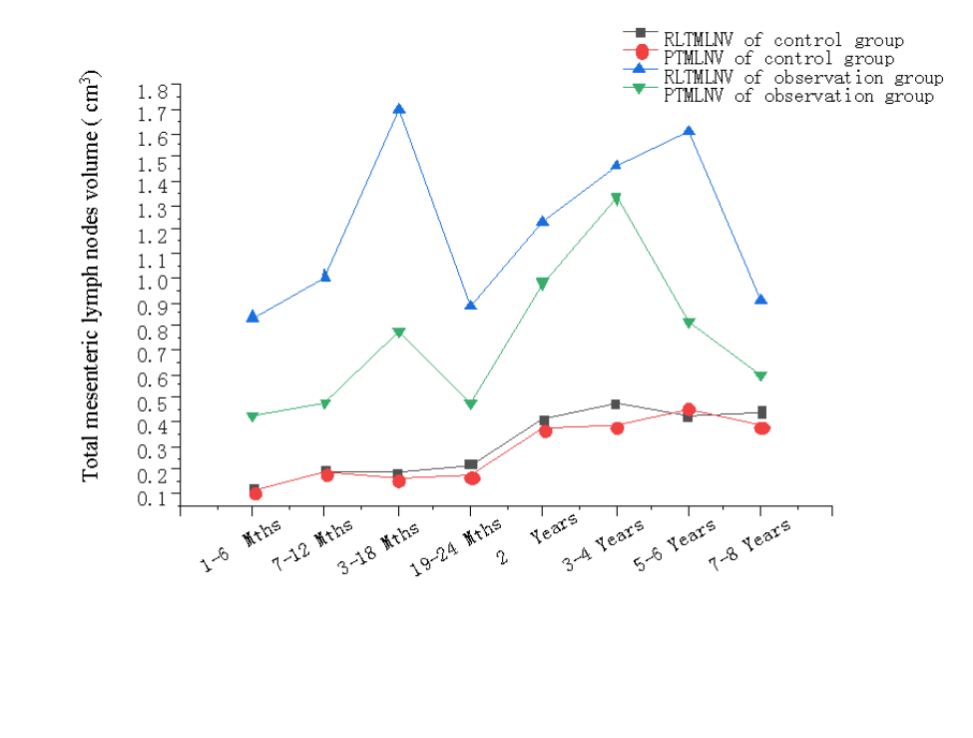
Mean values of RLTMLNV and PTMLNV at different age stages in the control and observation groups

In the control group, there was no significant difference between the RLTMLNV and PTMLNV in all age groups (P > 0.05) (Table 4). The mean value of RLTMLNV in the observation group was greater than that of PTMLNV(Fig. 1). Especially within 2 years of age, the mean value of RLTMLNV was significantly greater than that of PTMLNV in the observation group, with statistical significance (P < 0.05) (Table 4). This result suggests that the RLTMLNV is a better predictor of intussusception than PTMLNV, especially before 2 years of age (Fig. 1). The occurrence of intussusception is more closely related to the hyperplasia of intestinal lymphoid tissue in the right lower abdomen.

### The variation characteristics of RLTMLNV and PTMLNV in the control and observation groups at different age groups.

According to the various characteristics of RLTMLNV and PTMLNV in the control group and the observation group, the hyperplasia of intestinal lymph nodes in the right lower abdomen and periumbilical lymph nodes in healthy children was in a state of low hyperplasia before 2 years old, and was in a state of moderate hyperplasia and kept in a plateau period with little changes after 2 years old. There is a significant difference between these two age stages(P = 0.001) (Fig. 1). While in children with intussusception, the hyperplasia of intestinal-related lymphoid tissue in the right lower abdomen and the periumbilical region was severe hyperplasia at all age stages. There was no significant difference in the hyperplasia of mesenteric lymphoid tissue in the right lower abdomen among all age groups (P = 0.834) (Fig. 1), and there was also no significant difference in the hyperplasia of mesenteric lymphoid tissue at the periumbilical region among all age groups (P = 0.097) (Fig. 1).

### Comparison of the receiver operating characteristic curve (ROC) value in predicting intussusception between RLTMLNV and PTMLNV.

**Fig. 2:**
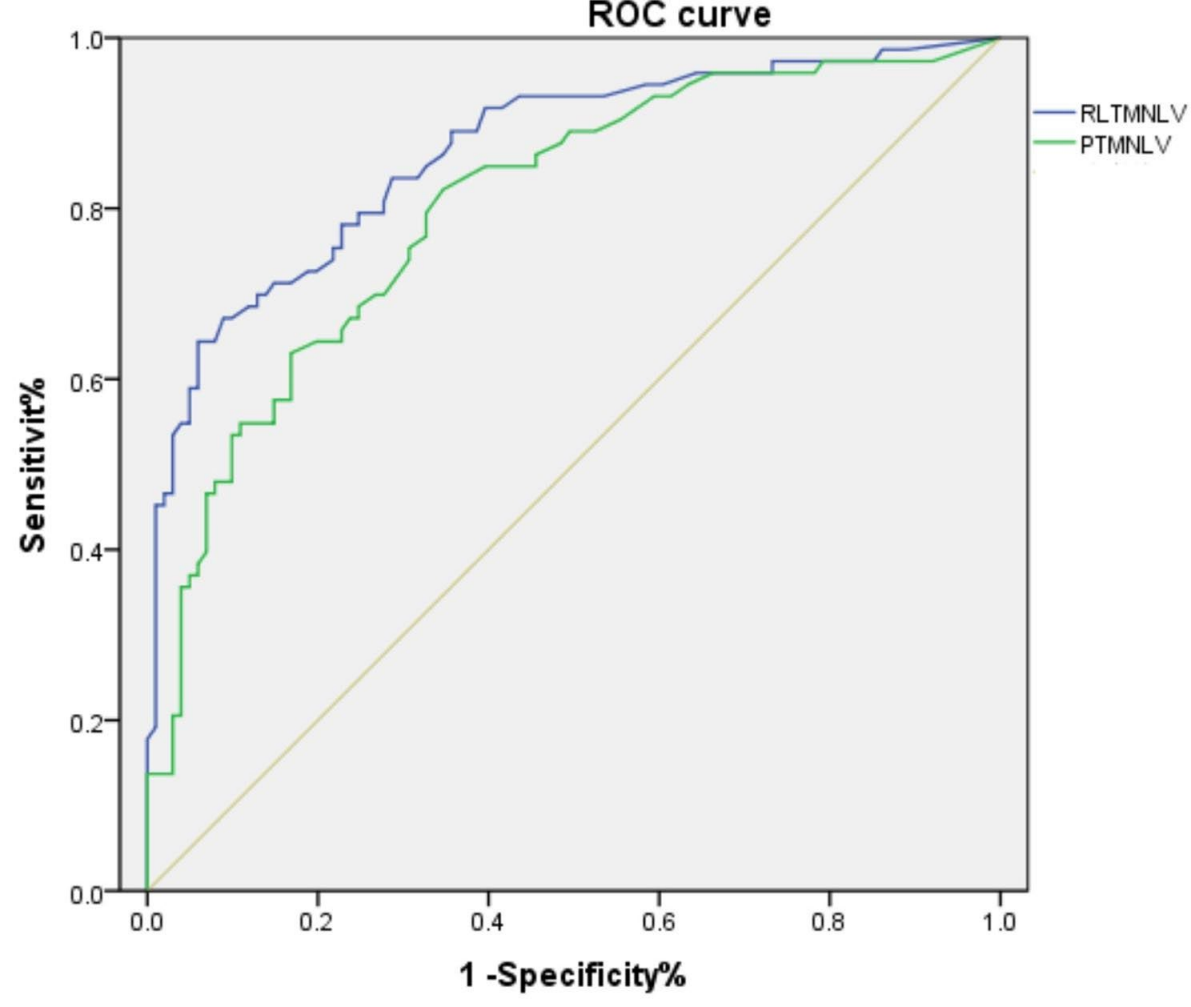
Predictive ROC curves of RLTMLNV and PTMLNV for intussusception in children

The receiver operating characteristic curve (ROC) was used to establish the predictive curves of RLTMLNV and PTMLNV for children with or without intussusception (Fig. 2). The area under the ROC curve (AUC) of the RLTMLNV was 0.866 (95% confidence interval was 0.81–0.922), and the optimal cut-off value of the RLTMLNV for predicting intussusception was 0.78cm^3^, with a sensitivity of 64.4% and specificity of 94.1%. The area under the ROC curve (AUC) of the PTMLNV was 0.802 (95% confidence interval was 0.735–0.868), and the optimal cut-off value of the PTMLNV for predicting intussusception was 0.29 cm^3^, with a sensitivity of 82.2% and specificity of 65.3%.

**Table 1:** Cases of different age groups in the control group and observation group (n)

Grouping	1–6 Mth	7–12 Mth	13-18Mth	19–24 Mth	2Year	3–4 Year	5–6 Year	7–8 Year	Total
Control group	18	11	10	7	10	14	16	15	101
Observation group	4	15	10	10	10	15	6	3	73

**Table 2:** Median(interquartile range) of RLTMLNV in different age groups of the control group and the observation group( cm3 ), and the difference comparison between them

Grouping	1–6 Mth	7–12 Mth	13–18 Mth	19–24 Mth	2 Year	3–4 Year	5–6 Year	7–8 Year
Control group	0.11(0.14)	0.19(0.28)	0.19(0.3)	0.15(0.41)	0.46(0.6)	0.39(0.59)	0.34(0.39)	0.31(0.35)
Observation group	0.82(0.08)	0.86(1.19)	1.8(1.9)	0.9(0.64)	1.18(1.62)	1.37(1.85)	0.84(2.6)	0.83(0)
P value	0.002	0.001	0.001	0.013	0.023	0.015	0.010	0.038

**Table 3:** Median(interquartile range) of PTMLNV in different age groups of the control group and the observation group(cm3), and the difference comparison between them

Grouping	1–6 Mth	7–12 Mth	13–18 Mth	19–24 Mth	2 Year	3–4 Year	5–6 Year	7–8 Year
Control group	0.11(0.15)	0.13(0.28)	0.12(0.24)	0.07(0.32)	0.21(0.53)	0.29(0.18)	0.45(0.31)	0.27(0.37)
Observation group	0.38(0.22)	0.43(0.39)	0.74(0.87)	0.43(0.37)	0.82(0.8)	0.88(1.45)	0.74(0.68)	0.65(0)
P value	0.002	0.007	0.002	0.032	0.008	0.011	0.105	0.086

**Table 4:** Comparison of the differences between the RLTMLNV and PTMLNV in all age groups of the control group and observationgroup

Grouping	1–6 Mth	7-12Mth	13–18 Mth	19–24 Mth	2 Year	3–4 Year	5–6 Year	7–8 Year
P value of control group	0.605	0.508	0.515	0.612	0.799	0.638	0.569	0.776
P value of observation group	0.068	0.005	0.007	0.028	0.445	0.460	0.173	0.285

## Discussion

From the perspective of immunology [4], children’s immune system gradually begins to be exposed to various foreign antigens, including viruses, bacteria, and other significant molecular antigen substances, after birth. The respiratory tract, digestive tract and skin are the main parts of the human body exposed to various foreign antigens. Exposure of the child’s immune system to antigens can lead to the proliferation of B-cell clones and T-cell clones that are specific to foreign antigens, resulting in the proliferation of lymphoid tissue and lymph nodes in the corresponding surrounding area. Antigenic substances from the respiratory tract can cause tonsil enlargement, adenoidal hypertrophy, and cervical lymph node hyperplasia. Similarly, intestinal antigens can cause lymphocyte hyperplasias, such as submucosal lymphoid hyperplasia of the intestinal wall and mesenteric lymph node enlargement. Lymphoid hyperplasia is a normal or abnormal pathophysiological reaction after the immune system’s contact with foreign antigens. The proliferation of lymphoid tissue produces many B lymphocytes that can produce specific antibodies and T lymphocytes. So that the immune ability of children immune system is gradually enhanced and improved. With the gradual increase of children’s age and the enhancement of immunity, the opportunity for foreign antigens to enter the body is gradually reduced, or the generation of immune tolerance, this lymphoid tissue hyperplasia may gradually reduce or even disappear. The degree of proliferation depends on the type and quantity of antigens and the proliferative ability of individual lymphocytes. Mild lymphoid hyperplasia will not cause particular discomfort to children, while severe lymphoid hyperplasia may cause some obstructive lesions and sometimes requires surgical treatment [10–12].

The hyperplasia of submucosal lymphoid tissue at the distal ileum resulted in nodular protrusion of intestinal mucosa into the intestinal lumen and ileocecal valve hypertrophy. In addition, children’s intestinal lumen is relatively thin, and the tubercle protrusion is relatively large compared with the small intestinal lumen diameter. Children’s intestinal peristalsis is faster than adults, and the intestinal wall is thinner and more plastic. These tubercle protrusions can pull the intestinal wall of the terminal ileum to enter the ascending colon, causing intussusception.

Lymphoid tissue in the intestinal wall of children includes lymphatic nodules and diffuse lymphoid tissue in the lamina propria and submucosa, among which the distal ileum and appendix are the most abundant lymphoid tissue. So the distal ileum is also the site most prone to lymphoid tissue hyperplasia [13, 14]. However, lymphoid hyperplasia of the terminal ileum is difficult to detect with the existing non-invasive clinical examination techniques, including ultrasound, endoscopy, and X-ray barium meal fluoroscopy. Even in laparotomy, it is difficult to detect the hyperplasia of submucosal lymphoid tissue at the distal ileum from the appearance and touch of the hand without opening the intestine. Therefore, finding an indirect indicator of submucosal lymphoid tissue proliferation of the intestinal wall is necessary.

The total volume of mesenteric lymph nodes`(RLTMLNV and PTMLNV), which are easily detected by ultrasound, is just an ideal indicator. The reason is that mesenteric lymph nodes and intestinal wall submucosal lymphoid tissue are stimulated by the same antigens in the intestinal cavity and cause lymphoid tissue hyperplasia in most cases, so mesenteric lymph node hyperplasia and intestinal submucosal lymphoid tissue hyperplasia have the synchronization. Thus, we can indirectly predict the degree of submucosal lymphoid hyperplasia of the intestinal wall by measuring the degree of hyperplasia of nearby mesenteric lymph nodes. In addition, the right lower abdominal mesenteric lymph node mainly drains lymphocytes and intestinal antigens of the ileocecal intestine, and the periumbilical mesenteric lymph node mainly drains intestinal antigens and lymphocytes of other parts of the intestine. So, lymph node hyperplasia in the right lower abdominal mesentery can better reflect the lymphoid hyperplasia in the ileocecal intestinal wall. Moreover, the number and size of mesenteric lymph nodes can be easily detected by ultrasound, and a simple calculation can calculate the total volume of mesenteric lymph nodes.

Our results showed that the mesenteric lymph nodes in the observation group were severely hyperplastic, and the total volume of mesenteric lymph nodes in the right lower abdomen and periumbilical mesenteric lymph nodes in the observation group were significantly larger than those in the control group, with significant differences (P = 0.001) (Tables 2 and 3). With the increase of age, the P value showed an increasing trend (Tables 2 and 3). This result shows that the incidence of intussusception is closely related to intestinal-associated lymphoid hyperplasia (mesenteric lymphoid tissue + intestinal wall lymphoid tissue). The relationship between intussusception and intestinal lymphoid hyperplasia in elderly children (> 4 years old) decreased, and the P value gradually increased (Table 3). The possibility of intussusception induced by other factors or organic lesions increased.

Table 4 shows no significant difference in lymphoid tissue hyperplasia between the right lower abdominal area and the periumbilical region in the control group at different ages. In the observation group, the proliferation of mesenteric lymphoid tissue was more evident in the right lower abdomen than in the periumbilical mesenteric lymphoid tissue. The mean value of RLTMLNV in the observation group, especially within 2 years old, was significantly higher than that of PTMLNV (P < 0.05). After 2 years of age, the degree of proliferation of mesenteric lymphoid tissue in the observation group around the umbilicus and right lower abdomen gradually approached, and there was no significant difference during the period (P > 0.05) (Table 4). This result suggests that RLTMLNV is a better predictor of intussusception than PTMLNV, mainly before 2 years of age (Fig. 1). The occurrence of intussusception is more closely related to the proliferation of intestinal-associated lymphoid tissue in the right lower abdomen.

In most normal children (control group), the right lower abdominal and periumbilical mesenteric lymph nodes showed low-grade hyperplasia at the age of < 2 years and moderate hyperplasia at the age of 2–8 years, with significant differences between these two age stages (P = 0.001) (Fig. 1). The children with intussusception (observation group) showed severe hyperplasia of gut-associated lymphoid tissue at all ages in the right lower abdomen and around the umbilicus. There was no significant difference in the mesenteric lymphoid tissue hyperplasia among all age groups in the right lower abdomen (P = 0.834). There was also no significant difference in the mesenteric lymphoid tissue hyperplasia among all age groups around the umbilicus (P = 0.097). Compared with the control group, the children with intussusception had severe mesenteric lymphoid hyperplasia at all ages, and there was no significant difference in the degree of hyperplasia among all age groups (Fig. 1).

The receiver operating characteristic curve (ROC) was used to analyze RLTMLNV and PTMLNV ( Fig. 2). The area under the ROC curve (AUC) of RLTMLNV was 0.866 (95% confidence interval was 0.81–0.922), the cut-off value was 0.78cm3, the sensitivity was 64.4%, and the specificity was 94.1%. The area under the ROC curve (AUC) of PTMLNV was 0.802 (95% confidence interval 0.735–0.868), the cut-off value was 0.29 cm3, the sensitivity was 82.2%, and the specificity was 65.3%. PLTMLNV has a better predictive value for intussusception than PTMLNV.

## Conclusion

These results suggest intestinal-associated lymphoid hyperplasia is an important cause of intussusception, especially submucosal lymphoid hyperplasia at the distal ileum. Hyperplasia of lymphoid tissue in the submucosa and ileocecal valve of the terminal ileum forms traction points, which draw the distal ileum into the ascending colon to induce intussusception.

## Data Availability

All data and materials are included in the article.

## Abbreviations

RLTMLNV: right lower abdominal total mesenteric lymph node volume
PTMLNV: periumbilical total mesenteric lymph node volume
MALT: mucosal-associated lymphoid tissue
HEV: high columnar endothelial venule.
